# Investigation and identification of protein carbonylation sites based on position-specific amino acid composition and physicochemical features

**DOI:** 10.1186/s12859-017-1472-8

**Published:** 2017-03-14

**Authors:** Shun-Long Weng, Kai-Yao Huang, Fergie Joanda Kaunang, Chien-Hsun Huang, Hui-Ju Kao, Tzu-Hao Chang, Hsin-Yao Wang, Jang-Jih Lu, Tzong-Yi Lee

**Affiliations:** 1 Department of Obstetrics and Gynecology, Hsinchu Mackay Memorial Hospital, Hsin-Chu, 300 Taiwan; 2 Mackay Medicine, Nursing and Management College, Taipei, 112 Taiwan; 3 Department of Medicine, Mackay Medical College, New Taipei City, 252 Taiwan; 4 Department of Medical Research, Hsinchu Mackay Memorial Hospital, Hsin-Chu, 300 Taiwan; 5 Department of Computer Science and Engineering, Yuan Ze University, Taoyuan, 320 Taiwan; 6 Tao-Yuan Hospital, Ministry of Health & Welfare, Taoyuan, 320 Taiwan; 7 Graduate Institute of Biomedical Informatics, Taipei Medical University, Taipei, 110 Taiwan; 8 Department of Laboratory Medicine, Chang Gung Memorial Hospital at Linkou, Taoyuan, 333 Taiwan; 9 Department of Medical Biotechnology and Laboratory Science, Chang Gung University, Taoyuan, 333 Taiwan; 10 Innovation Center for Big Data and Digital Convergence, Yuan Ze University, Taoyuan, 320 Taiwan

**Keywords:** Reactive Oxygen Species (ROS), Protein carbonylation, Amino acid composition, Physicochemical properties

## Abstract

**Background:**

Protein carbonylation, an irreversible and non-enzymatic post-translational modification (PTM), is often used as a marker of oxidative stress. When reactive oxygen species (ROS) oxidized the amino acid side chains, carbonyl (CO) groups are produced especially on Lysine (K), Arginine (R), Threonine (T), and Proline (P). Nevertheless, due to the lack of information about the carbonylated substrate specificity, we were encouraged to develop a systematic method for a comprehensive investigation of protein carbonylation sites.

**Results:**

After the removal of redundant data from multipe carbonylation-related articles, totally 226 carbonylated proteins in human are regarded as training dataset, which consisted of 307, 126, 128, and 129 carbonylation sites for K, R, T and P residues, respectively. To identify the useful features in predicting carbonylation sites, the linear amino acid sequence was adopted not only to build up the predictive model from training dataset, but also to compare the effectiveness of prediction with other types of features including amino acid composition (AAC), amino acid pair composition (AAPC), position-specific scoring matrix (PSSM), positional weighted matrix (PWM), solvent-accessible surface area (ASA), and physicochemical properties. The investigation of position-specific amino acid composition revealed that the positively charged amino acids (K and R) are remarkably enriched surrounding the carbonylated sites, which may play a functional role in discriminating between carbonylation and non-carbonylation sites. A variety of predictive models were built using various features and three different machine learning methods. Based on the evaluation by five-fold cross-validation, the models trained with PWM feature could provide better sensitivity in the positive training dataset, while the models trained with AAindex feature achieved higher specificity in the negative training dataset. Additionally, the model trained using hybrid features, including PWM, AAC and AAindex, obtained best MCC values of 0.432, 0.472, 0.443 and 0.467 on K, R, T and P residues, respectively.

**Conclusion:**

When comparing to an existing prediction tool, the selected models trained with hybrid features provided a promising accuracy on an independent testing dataset. In short, this work not only characterized the carbonylated substrate preference, but also demonstrated that the proposed method could provide a feasible means for accelerating preliminary discovery of protein carbonylation.

**Electronic supplementary material:**

The online version of this article (doi:10.1186/s12859-017-1472-8) contains supplementary material, which is available to authorized users.

## Background

Post-translational modifications (PTMs) are involving the attachment of chemical groups on a specific residue of proteins, which play significant roles in regulating many cellular processes such as differentiation of cell, protein degradation, processes of signaling and regulatory, regulation of gene expression, and protein-protein interactions [1, 2]. Enzymes catalyzed the attachment and removal of chemical groups for proteins. For example, protein phosphorylation is catalyzed by kinases in a signaling cascade and can be removed the phosphate by a phosphatase [3]. In other word, most PTMs are enzymatically controlled and regulated in cellular processes. Interestingly, there are PTMs that occur in a non-regulated manner which often caused by the structural features of proteins, the environments, and by the generation of free radicals surrounding the proteins. These kinds of PTMs are known as non-enzymatic protein modifications. There are some types of PTMs that are non-enzymatically occurred including oxidation, racemization, dityrosine, chloronitrotyrosine, isomerization, deamidation, nitration, carbonylation, carbamylation, and glycation (or glycoxidation) [4, 5]. Reduction of sequential electron of molecular oxygen establishes reactive oxygen species (ROS). It had been examined that reactive oxygen species had non-particularly and indistinguishably react with biomolecules as well as lipids, DNA, proteins, and small molecules [6]. ROS can modify and damage these biomolecules through oxidation resulting in oxidative stress [6, 7] and lead to the loss of proteins function (enzymatic activity) [8]. However, the generation of oxidative damage on cells mostly happen on proteins for they are often catalysts rather than stoichiometric mediators [9].

Small amount of ROS are important in signaling pathways and in the resistance toward violating pathogens [7]. Oxidative stress occurs when the amount of ROS are highly produced and surpass the cell’s ability to detoxify them [7, 10]. Oxidative stress can cause various kinds of PTMs including hydroxylation, nitration, sulfhydrylation, carbonylation, and glutathionylation [8]. Carbonylation is an irreversible protein modification and has been used as the biological marker of oxidative stress because of its relative stability, early formation, and the availability of analytical strategies to quantify it compared to another oxidative stress induced protein modifications [8, 9]. Protein carbonylation typically involve three manners (Additional file 1: Figure S1): the first one is by direct oxidation with ROS on amino acid side chains of Lysine (K), Arginine (R), Threonine (T), and Proline (P) resulting in carbonyl derivatives of 2-pyrrolidone from proline, glutamic semialdehyde from arginine and proline, α-aminoadipic semialdehyde from lysine, and 2-amino-3-ketobutyric acid from threonine; the second one is through Michael addition reaction of α,β-unsaturated aldehydes derived from lipid peroxidation; the last one is by addition of reactive carbonyl derivatives (ketoamines, ketoaldehydes, deoxyosones) as the production of the reaction of reducing sugars or their oxidation products with the amino group of lysine residue (glycation and glycoxidation reactions) which yield the advance glycation end products (AGEs) [7, 9, 11]. In protein carbonylation, various mechanisms altered the side groups of K, R, T and Pro residues including metal-catalyzed oxidation (MCO) [12]. As a consequence of oxidative modifications, protein carbonylation has been associated with several age-related or metabolic diseases such as Alzheimer, Parkinson, Diabetes, Chronic lung disease, etc. [5, 7, 8].

Several experimental assays such as spectrophotometric, enzyme-linked immunosorbent, slot blotting have been employed to experimentally identify carbonylation sites [9]. Additionally, mass spectrometry-based proteomics [13, 14] have been used for site-specific identification of carbonylated peptides. Due to the labile nature of the ROS bond and the low abundance of endogenously carbonylated proteins in vivo, however, the unambiguous identification of carbonylated proteins and modified sites remains challenging by commonly used proteomic technology. From the view point of substrate site specificity, thus, it is important to develop a systematic method for the comprehensive investigation efficient of protein carbonylation sites. As listed in Additional file 2: Table S1, Maisonneuve et al. developed a computational analysis tool named CSPD evaluated using jackknife testing, to detect the carbonylation sites of Escherichia coli proteome [15]. Another prediction tool named CarsPred was developed to predict the carbonylation sites on human proteins using WSVM with 10-fold cross-validation [16].

With the limited information about protein carbonylation, this work provides a full characterization of carbonylated substrate sites based on various features, including linear amino acid sequences and physicochemical properties. In this investigation, totally seven types of features, such as amino acid sequence (AA), amino acid composition (AAC), amino acid pair composition (AAPC), positional weighted matrix (PWM), position specific scoring matrix (PSSM), accessible surface area (ASA), and the physicochemical properties of proteins, were examined. To test the predictive power of those examined features in identifying carbonylation sites, three classifiers, namely support vector machine (SVM), decision tree (DT) and random forest (RF), were employed to build up the predictive models using each type of feature. Additionally, the combination of hybrid features was also considered for improving the predictive performance, based on the evaluation of five-fold cross-validation. Finally, an independent testing dataset, which is truly blind to the process of model construction, was obtained from research articles and was applied to further evaluate the effectiveness of the chosen model on the testing data from multiple species.

## Methods

### Data collection and preprocessing

**Fig. 1:**
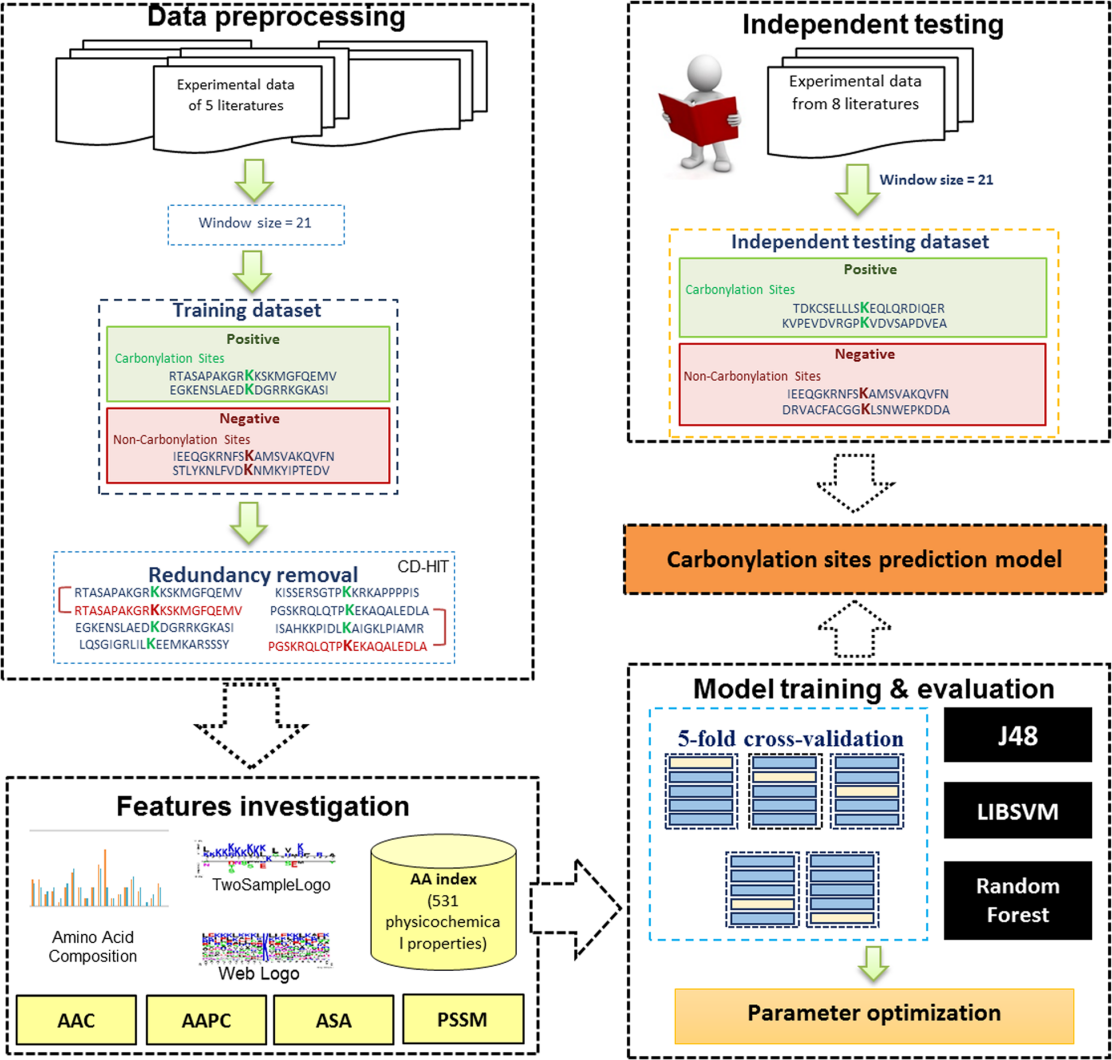
Analytical flowchart of the identification of protein carbonylation sites

A majority of the experimental data used in this study was obtained from literatures, which comprised site-specific information on experimentally confirmed carbonylated peptides in humans. The analytical flowchart of this work is depicted in Fig. 1. Without the public database available for protein carbonylation, the dataset used in this investigation was obtained from five literatures [17–21], which is similar with the training dataset used in CarsPred [16]. Detailed statistics of these five data resources are provided in Additional file 3: Table S2. After the removal of redundant data, totally 226 non-redundant carbonylated proteins in human are regarded as training dataset, which comprised 307, 126, 128, and 129 carbonylation sites for K, R, T and P residues, respectively. To construct the positive training dataset (carbonylated sites), the window length of 2*n* + 1 was employed to extract sequence fragments centering at the experimentally verified carbonylation sites as well as containing *n* upstream and *n* downstream flanking amino acids. Carbonylated sites in the KRTP-enriched region was set to 4 residues long and considered in KRTP-enriched region if it contained 3 local enrichment of K, R, T, or P [15]. On the other hand, given 226 experimentally verified ubiquitinated proteins, the sequence fragments containing window length of 2*n* + 1 amino acids and centering at K, R, T and P residues without the annotation of carbonylation were regarded as the negative training dataset (non-carbonylation sites). Based on the overall prediction performance of a previous work [16] and our preliminary evaluation by using various window lengths, the window size of 21 (*n* = 10) provides an effective and stable accuracy in the identification of carbonylation sites on four residues. By using a window size of 21, consequently, the negative training dataset contained 2577, 912, 1211 and 1317 sequences on K, R, T and P residues, respectively.

In order to elude the overestimation of predictive performance in this work, CD-HIT [22] program was applied to remove homologous sequences from the training dataset. Due to the limited annotation of protein carbonylation sites, based on the analysis of sequence fragments, it would be possible that some negative data are identical with positive data in the training dataset, potentially resulting in false positive or false negative predictions. Hence, CD-HIT was further applied by running cd-hit-2d across positive and negative training dataset with 100% sequence identity, to solve this problem. After having filtered out homologous fragments with 50% sequence identity (by running cd-hit and psi-cd-hit), Table 1 shows that the final training dataset consisted of 206, 101, 96, and 94 positive sequences on K, R, T, and P, respectively. Additionally, the final training dataset is composed of 1166, 504, 488, and 412 and 5176 negative sequences on K, R, T, and P, respectively.

**Table 1:** Data statistics of positive and negative sequences (with window size 21) in training and testing datasets

Dataset	Residues	Number of proteins	Number of positive sequences	Number of negative sequences
Training dataset	K	155	206	1166
	R	90	101	504
	T	81	96	488
	P	77	94	412
Independent testing dataset	K	67	78	301
	R	65	67	276
	T	50	53	124
	P	71	82	304

In the binary classification of carbonylation sites, the constructed model might be overfitted to the training dataset and thus perform highsometimes With an attempt to evaluate the real performance of the constructed models, we further generate an independent testing dataset, which was definitely blind to the training dataset. The dataset for independent testing was manually extracted from seven literatures [15, 17, 18, 23–26], which comprised 117, 90, 62, and 104 carbonylation sites, respectively, on K, R, T and P residues from multiple species. The positive and negative testing data were constructed using the same approach as was applied to the training dataset. Besides, the program cd-hit-2d, using sequence identity cutoff at 100%, was applied again to remove the data redundancy between independent testing dataset and training dataset. This resulted in the final independent testing dataset consisting of 78, 67, 53 and 82 positive sequences on K, R, T and P, respectively (Table 1). In addition, the negative dataset for independent testing is composed of 301, 276, 124 and 304 sequences on K, R, T, and P, respectively. Moreover, the testing dataset was also used to evaluate the predictive power of other prediction tools, which were compared with the presented method in terms of predictive performance.

### Features investigation

In this study, numerous sequence-based features, including amino acid sequence, amino acid composition (AAC), amino acid pair composition (AAPC), positional weighted matrix (PWM), position-specific scoring matrix (PSSM), solvent-accessible surface area (ASA) and physicochemical properties (AAindex), were assessed to conduct the best prediction model. After the extraction of sequence fragments with a window size of 21-mer amino acids, each sequence fragment was encoded based on the investigated features. For a binary classification, the labels +1 and -1 were corresponded to the positive and negative training data, respectively.

### Amino acid sequence (AA)

The orthogonal binary coding mechanism is one of the most popular coding methods for transforming amino acid sequence into a numeric vector, called 20-dimensional binary coding [27]. Each amino acid was represented by a vector with 20 letters. For example, alanine (A) would be encoded as “10000000000000000000” while cysteine (C) would be “01000000000000000000” and so on. This coding scheme could unify the distance among twenty types of amino acids. For each sequence fragment, the length of feature vectors with a window size of 2*n* + 1 was set to (2*n* + 1) × 20 to represent the flanking amino acids surrounding the carbonylation or non-carbonylation sites. Therefore, there were a total of k vectors {x_i_, *i* = 1, 2 …, *k*} corresponding to the number of *k* sequence fragments in the training and testing datasets.

### Amino acid composition (AAC)

Amino acid composition is a common feature for generating predictive model, involving the composition of the frequency of each amino acid residue inside the protein sequence. For a protein sequence *n*, let *f*_*x*_ represents the occurrence frequencies of its 20 native amino acids. Thus, the composition of amino acids *Px* is calculated by [28].

1 $$ Px\ (n) = \frac{f_x(n)}{{\displaystyle {\sum}_{i=1}^{20}}{f}_i(n)}\kern1em i,\ x=1,2, \dots,\ 20 $$

Then the composition of protein *n* is then defined as

2 $$ P(n) = \left[{p}_1(n),{p}_2(n),\dots,\ {p}_{20}(n)\right] $$

### Amino Acid Pair Composition (AAPC)

Another composition of amino acids introduced by Park [29] is amino acid pair composition (AAPC): each sequence fragment in the training dataset is represented by a vector {*x*_*i*_, *i* = 1,…,*n*}, where vector *x*_*i*_ has 400 elements for the amino acid dipeptide composition. For the coding of amino acid dipeptide composition, the 400 elements specify the numbers of occurrences of 400 amino acid dipeptides normalized with the total number of dipeptides in a sequence fragment. In further exploring potential features for protein classification, various methods aimed at selecting relevant sequence features given a large set of features have been used [30]. In this work, the importance of amino acid pairs in identifying splicing factors is further investigated by means of measuring the statistical significance of each dipeptide in the data set. For each amino acid pair, the number of positive and negative sequences containing the target dipeptide is calculated separately. The statistical significance of each dipeptide is then obtained by examining a sample against a background set based on the hypergeometric equation (*P*-value) [31]:

3 $$ P(t)={\displaystyle \sum_t^T\frac{C_t^T\cdot {C}_{k-t}^{K-T}}{C_k^K}} $$

where *K* is the background set represented by the number of all protein sequences and *T* is the sample set represented by the number of positive sequences; *k* is the number of all proteins having the target amino acid pair and *t* is the number of positive sequences having the target amino acid pair. *P*-value is calculated for each dipeptide based on the hypergeometric equation. A smaller *p*-value corresponds to a greater statistical significance. Furthermore, the positive and negative probabilities of each amino acid pair are computed by means of dividing the number of positive sequences or negative sequences having the target amino acid pair by the total number of positive sequences or negative sequences, respectively. The probability difference between the positive and negative datasets is then obtained. In this work, amino acid pairs having a *p*-value less than 0.05 and a probability difference greater than 0 is considered as statistically informative for the identification of carbonylation sites.

### Positional weighted matrix (PWM)

With reference to the coding scheme in SulfoSite [32], the positional weighted matrix (PWM) of amino acids was determined using the positive training data. The coding scheme of PWM has been intensively adopted in various PTM prediction methods [27, 33–42]. The PWM described the frequency of occurrence of amino acids surrounding the carbonylation sites, and was utilized to encode the sequence fragments. Each residue in the training dataset was represented by a matrix of *m* × *w* elements, where the window size of 21 was designated by *w* and the 21 elements including the 20 types of amino acids as well as the terminal signal was denoted by *m*.

### Position-specific scoring matrix (PSSM)

Two proteins may share similar structures with different amino acid compositions so that several amino acid residues of a protein might be mutated without changing its structure and function [43]. In this work, evolutionary information of amino acids around the carbonylation sites is obtained using position-specific scoring matrix (PSSM). The PSSM profiles of each carbonylated protein were obtained by using PSI-BLAST [44] search against the non-redundant database of protein sequences compiled by NCBI [45]. Due to the fact that the data consists of protein sequences with variable length, a weighted scoring matrix is determined by summing up the position-specific scores of the same amino acids occurring in a protein sequence to get a uniform number of features. Additional file 4: Figure S2 displays the flowchart for generating a 400-dimensional (20 × 20 residue pairs) vector of each training sequence based on the PSSM profile, which is a matrix of *m* × 20 elements where *m* represents the protein sequence length and 20 represents the position specific scores for each type of amino acid. Then, the PSSM profile is transformed to a 20 × 20 matrix by summing up each row of same amino acid in the PSSM profile. Finally, every element of 400-dimensional PSSM vector is divided by the length of the sequence and then is normalized by $\frac{1}{1+{e}^{-x}}$ for scaling the values between 0 and 1.

### Accessible surface area (ASA)

The solvent-accessible surface area (ASA), determining the accessibility of an amino acid side-chain on the surface of a protein that can be accessed by solvent, was also considered as an feature for identifying carbonylation sites. With the limited tertiary structures of carbonylated proteins in the Protein Data Bank (PDB) [46], the RVP-Net [47, 48] program was employed to compute the ASA value from the protein sequence. RVP-Net could compute the real ASAs of amino acids by using a neutral network approach with the consideration of amino acid composition in neighborhood. The possible mean absolute error, which was defined as the absolute difference between the predicted and experimental values of relative ASA per residue [48], was 18.0–19.5%, for each measurement. The value of ASA represented the percentage of the solvent-accessible area of each amino acid on the protein. Full-length carbonylated protein sequences were submitted into the RVP-Net for computing the ASA values of all of the residues. Then, the ASA values of amino acids surrounding the carbonylaed and non-carbonylated sites were extracted and normalized based on a scale from zero to one.

### Physicochemical properties

In order to explore physicochemical properties around the carbonylation sites, totally 544 amino acid indices were obtained from the AAindex [49] (Version 9.1), which specify the physicochemical properties of twenty amino acids. After the removal of amino acid indices containing the value “NA”, the remaining 531 physicochemical properties were examined to determine their ability to distinguish carbonylation sites from non-carbonylation sites. Given a specific physicochemical property in AAindex, a set of 20 numerical values was specified according to the evaluated physicochemical indices of the 20 amino acids. The sequence fragments were transformed from AAs surrounding carbonylated sites into values associated with their physicochemical properties. In order to identify the significant physicochemical properties, the F-score method [37, 40–43, 50, 51] was applied to compute a statistical value for each position surrounding carbonylation sites, based on the window length of 21. The F-score of the *i*th physicochemical feature is defined as:

4 $$ \mathrm{F}-\mathrm{score}(i)=\frac{{\left({{\overline{x}}_i}^{\left(+\right)}-{\overline{x}}_i\right)}^2+{\left({{\overline{x}}_i}^{\left(-\right)}-{\overline{x}}_i\right)}^2}{\frac{1}{n^{+}-1}{\displaystyle \sum_{k=1}^{n^{+}}{\left({x}_{k,i}^{\left(+\right)}-{{\overline{x}}_i}^{\left(+\right)}\right)}^2}+\frac{1}{n^{-}-1}{\displaystyle \sum_{k=1}^{n^{-}}{\left({x}_{k,i}^{\left(-\right)}-{{\overline{x}}_i}^{\left(-\right)}\right)}^2}} $$

where ${\overline{x}}_i$, ${{\overline{x}}_i}^{\left(+\right)}$ and ${{\overline{x}}_i}^{\left(-\right)}$ denote the average value of the *i*th feature in the whole, positive, and negative data sets, respectively; *n* ^+^ denotes the number of positive data set and *n* ^−^ denotes the number of negative data set; *x*_*k*,*i*_^(+)^ denotes the *i*th feature of the *k*th positive instance, and *x*_*k*,*i*_^(−)^ denotes the *i*th feature of the *k*th negative instance [52]. The performances of predictive models trained using the physicochemical properties individually were evaluated, and the properties were subsequently ranked in descending order based on the predictive accuracy.

### Combination of hybrid features

With an attempt to identify useful features for the prediction of protein carbonylation sites, the predictive power of each feature is evaluated based on cross-validation. Additionally, a hybrid approach is investigated in this work by combining different sets of feature vectors with the goal of improving predictive performance on the calssification between carbonylated and non-carbonylated sites. Prior to classification, the data needed to be scaled in the range of [-1, 1] to enhance the effectiveness of prediction [53]. For the construction of predictive models, hybrid features were generated by combining two or more single features. In order to obtain the highest predictive accuracy, the single features were selected based on the mRMR (minimum-redundancy maximum-relevance) [54] algorithm, which sorts the features according to their relevance to the target and the redundancy among the investigated features. The training feature with a smaller index implicates that it has a better trade-off between the maximum relevance and minimum redundancy [16]. The scoring function is defined as follows:

5 $$ scor{e}_j=I\left({f}_j,c\right)-\frac{1}{m}{\displaystyle \sum_{i=1}^mI\left({f}_i,{f}_j\right)} $$

where *f*_*j*_ ⊂ *S*_*n*_, *f*_*i*_ ⊂ *S*_*m*_, *S*_*m*_ = *S* − *S*_*n*_, and *S*_*m*_, *S*_*n*_, and *S* are the feature sets, as well as the *m* and *n* are the feature numbers. The mutual information *I(x,y)* is determined as follows:

6 $$ I\left(x,y\right)={\displaystyle \iint p\left(x,y\right) \log \frac{p\left(x,y\right)}{p(x)p(y)}\mathit{dxdy,}} $$

where *p(x,y)*, *p(x)*, and *p(y)* are the probabilistic density functions. In this investigation, a total of seven kinds of features, such as AA, AAC, AAPC, PWM, PSSM, ASA and AAindex, were ranked by mRMR criterion. Furthermore, the sequential forward selection (SFS), one of the typically used heuristic methods for feature selection, was adopted to determine the final combination of hybrid feature sets, based on the mRMR-ranking results. It involves the following steps:

1. *Use SVM, J48, or random forest as the classifier, and the five-fold cross-validation for predictive power estimate.*
2. *Select the first feature that has the best cross-validation performance among all features.*
3. *Select the feature, among all unselected features, combined with selected features that provide a better predictive performance.*
4. *Repeat the previous process until you have selected enough number of features, or until predictive performance is not improved anymore.*

### Construction of predictive models

#### Support Vector Machine (SVM)

One of the advanced machine learning method is Support Vector Machine (SVM) [55], which was intensively applied on pattern recognition and data classification. The positive and negative training datasets were used for building a predictive model with the identified support vectors. This binary classification utilizes a kernel function to transform the input samples into a higher dimensional space and attempts to find a hyper-plane to discriminate the two classes with maximal margin and minimal error. In our study, a public SVM library (LIBSVM) [56] was implemented to build models that could discriminate between carbonylation and non-carbonylation sites. In this work, the radial basis function (RBF) *K*(*S*_1_, *S*_*j*_) = exp(−*γ* ∥ *S*_*i*_ − *S*_*j*_ ∥ ^2^) was selected as the kernel function. Two factors were included to enhance the performance: the RBF kernel was determined by the gamma parameter, while the softness of the hyper-plane was modulated by the cost parameter. The range of the probability value set from 0 to 1 with LIBSVM library.

#### J48 decision tree (DT)

Decision tree (DT) is a tree-like model in which each internal node represents a “test” on an attribute, each branch represents the outcome of the test, and each leaf node represents a class label (positive or negative data) [57]. The path from root to a leaf node represents a descriptive rule containing conditional probabilities and possible consequences. J48 is an implementation of C4.5 decision tree algorithm using Java in WEKA data mining package. It is an improvement of ID3 algorithm which generates a decision tree with better effectiveness and efficiency. The constructed decision tree is then used as the model of the classification process and further employed to each tuple in the training dataset for yielding the predictive results [58]. In the construction of decision tree, the missing values are ignored by J48 program. For numeric attributes, the primary idea is to separate the numeric data into ranges based on the distribution of that attribute values in the training dataset [59].

#### Random forest (RF)

Random forest (RF) is a classifier proposed by Breiman L. [60], who delivers the ensemble of multiple classifiers based on randomly feature selection. Owing to its ability to supply an empirical approach to trail variable interactions, random forest is then considered as an appropriate classifier to handle large-scale dataset, especially for imbalanced dataset [61]. Random forest has been tested and used in many studies with a good result and be able to improve prediction accuracy as well as decrease the time consumption [61, 62]. In this study, a library of random forest program, integrated in WEKA data mining package, was adopted to construct the predictive model based on various features.

### Performance measurement

To examine the ability of the investigated features in identifying carbonylation sites, five-fold cross-validation was carried out for each feature to evaluate the predictive performance. The training dataset was divided into five subgroups with approximately equal size. The ratio of the testing set to the training set was 1:4 and the cross-validation process was repeated five times. The five validation results were then combined to generate a single estimation. Obviously, one of the benefits of k-fold cross-validation is the improvement on the reliability of evaluation because all of the original data, including the training and testing data sets, were considered and each subset should be tested only once. In this investigation, four measures such as sensitivity (Sn), specificity (Sp), accuracy (Acc), and Matthews Correlation Coefficient (MCC) were used:

7 $$ Sn = \frac{TP}{TP+FN} $$

8 $$ Sp = \frac{TN}{TN+FP} $$

9 $$ Acc = \frac{TP+TN}{TP+FP+TN+FN} $$

10 $$ MCC = \frac{\left(TP*\ TN\right) - \left(FN*FP\right)}{\sqrt{\left(TP+FN\right)*\left(TN+FP\right)*\left(TP+FP\right)*\left(TN+FN\right)}} $$

where TP, TN, FP and FN represented the number of true positives, true negatives, false positives and false negatives, respectively. The MCC reflects both the sensitivity (true positive rate) and specificity (true negative rate) of a predictive model. Sometimes, accuracy is not useful when the two classes are of very different sizes; hence, the MCC is usually regarded as a balanced measure even if the two classes are of very different sizes [35]. Finally, after selecting the best model with the highest MCC value, the independent testing dataset was used to test its real predictive power.

## Results and discussion

### Composition of amino acids around carbonylation sites

**Fig. 2:**
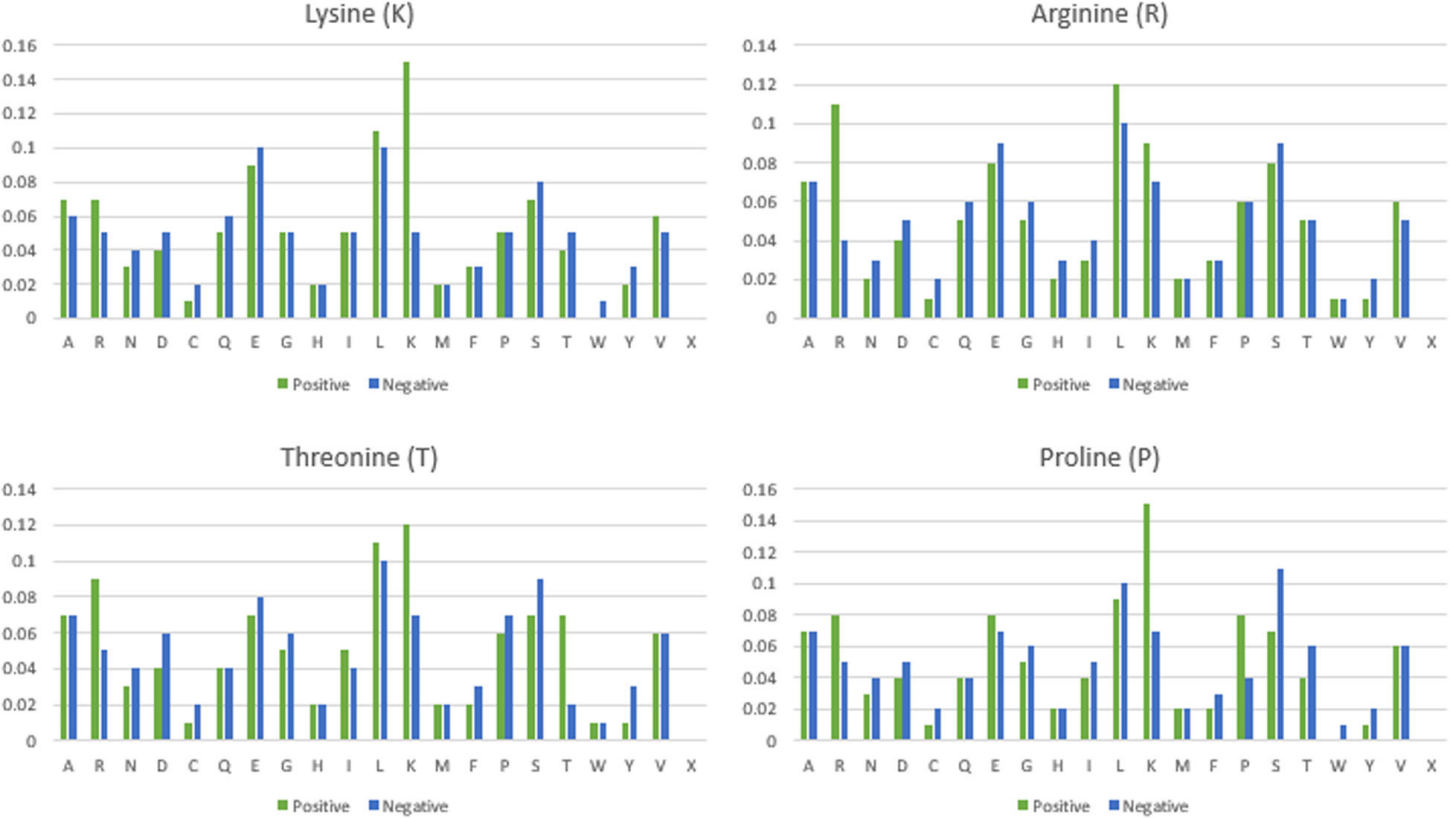
Comparison of amino acid composition between carbonylated and non-carbnylated sites on K, R, T and P residues

Based on the investigation of amino acid composition, the frequency of 20 amino acids around the carbonylated sites revealed the potential substrate environment for protein carbonylation. Figure 2 indicates that, at carbonylated lysines, K residue occur at a highest frequency surrounding the substrate sites, while C (Cysteine) and W (Tryptophan) residues have a relatively lower frequency of occurrence. For carbonylated arginines, R residue has a higher frequency in positive data compared to that in negative data. In addition, L (Leucine) and K residues are also relatively abundant around carbonylated arginines.

For carbonylated threonines, K, R and T residues are more abundant when comparing to non-carbonylated threonines. Lysine is also the most abundant amino acid around carbonylated prolines, while S (Serine) and T residues have a relatively lower frequency of occurrence.

**Fig. 3:**
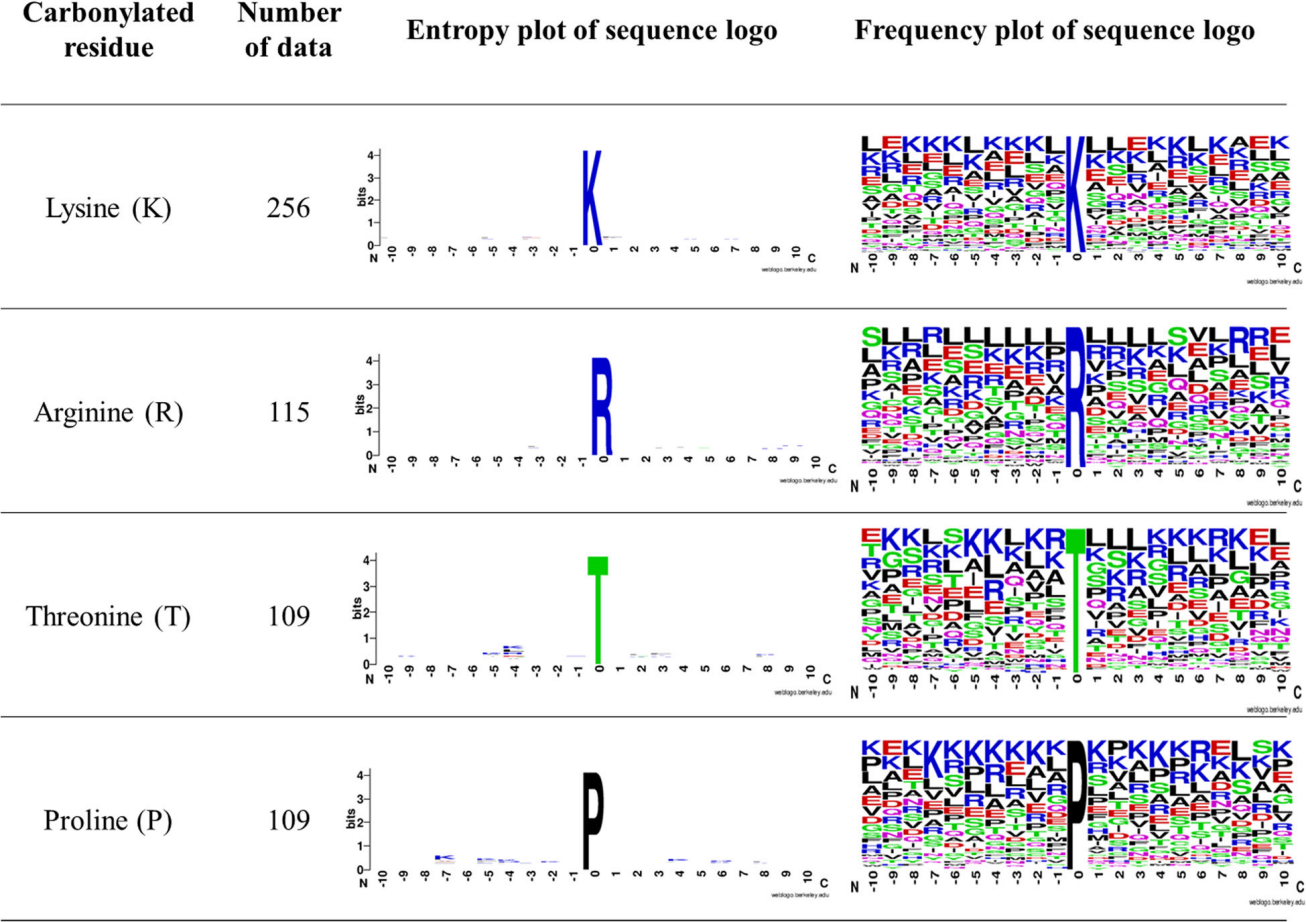
Entropy and frequency plots of position-specific amino acid composition of four carbonylated residues

**Fig. 4:**
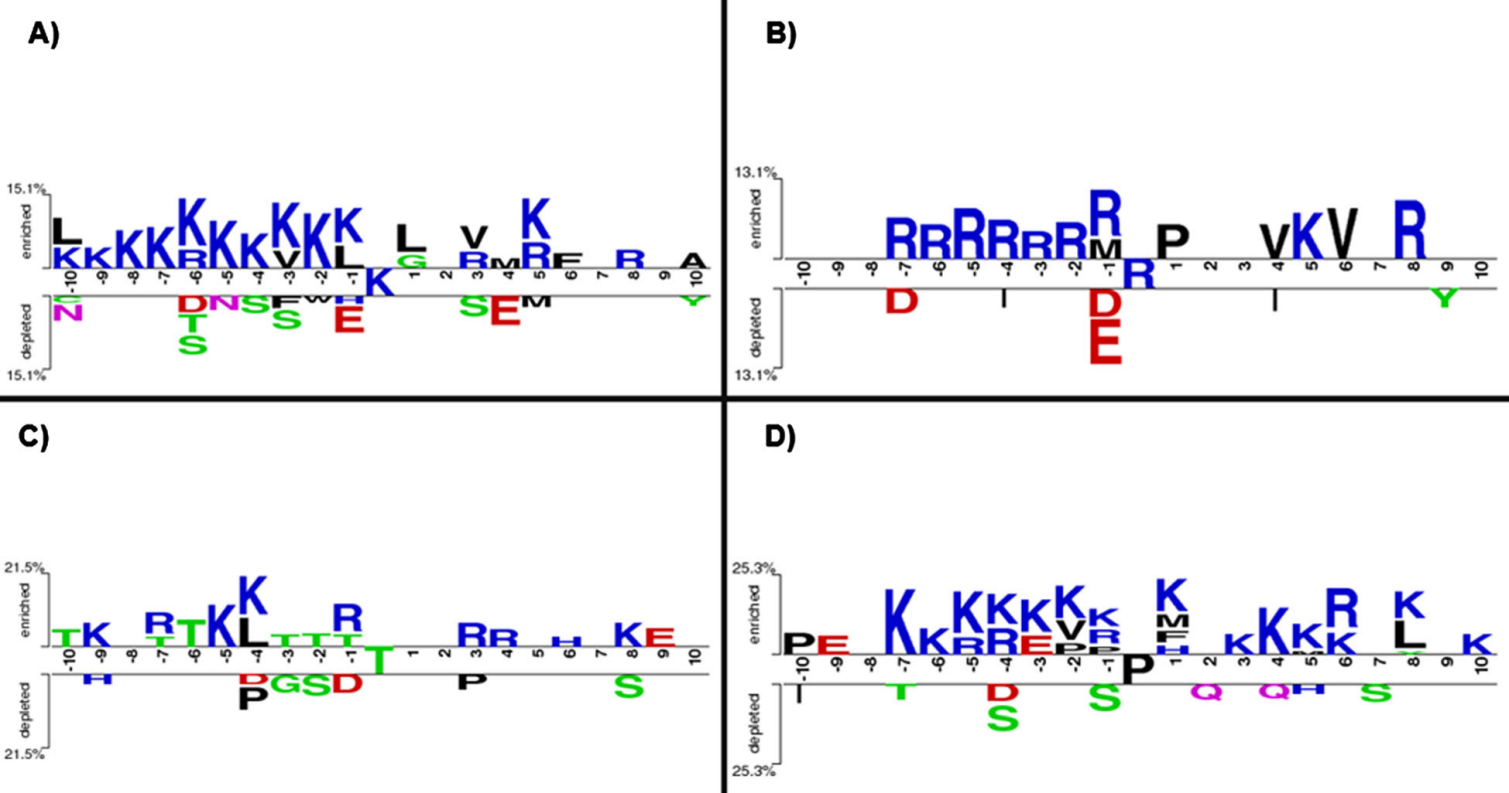
TwoSampleLogo of four carbonlated residues. **a** Two-Sample Logo of Lysine (K). **b** Two-Sample Logo of Arginine (R). **c** Two-Sample Logo of Thereonine (T). **d** Two-Sample Logo of Proline (P)

WebLogo [63] is usually used to generate the position-specific amino acid composition for the substrate sites of PTM, based on the training dataset. As presented in Fig. 3, both entropy and frequency plots of twenty amino acids at a specific position around carbonylation sites are generated base on the non-homologous positive training data. The entropy plots indicated that K and R residues (colored in blue) are slightly abundant around carbonylation sites. However, it is not trivial to identify the difference of amino acid composition between carbonylated and non-carbonylated sites position by position. To concentrate on notable differences between positive and negative sequences, TwoSampleLogo [64] was applied to calculate statistically significant enrichment and depletion in position-specific amino acid composition around carbonylation sites. Comparing between 206 positive and 1166 negative sequences (Fig. 4a), it was realized that three aforementioned amino acids (K, R and L) reach significant enrichment in the flanking region of carbonylation sites on lysine. In particular, the positively charged K and R residues had a significant enrichment at upstream region (from positions -10 to -1) with *p-value* < 0.01. Figure 4b implicated that the positively charged R residue is statistically enriched at upstream region (from positions -7 to -1) of carbonylated arginine residues. In contrast, at positions -1 that was close to carbonylated sites, a lack of negatively charged residues (D and E) was observed. Figure 4c showed that K, R and T residues are slightly enriched around the carbonylated threonine residues. Figure 4d also indicated that the positively charged K and R residues had a significant enrichment at both upstream (from positions -7 to -1) and downstream (from positions +1 to +10) regions with *p-value* < 0.01. The TwoSampleLogo results are consistent with the frequency of twenty amino acids around the carbonylated sites (as presented in Fig. 2). Additionally, it is clear that carbonylation sites are inclined to occur in KRTP-enriched regions which is conformable in Maisonneuve [15] and Rao’s [65] studies. This investigation also indicated that the positions of amino acids relative to one another in the sequence play a vital role in discriminating between carbonylated and non-carbonylated sites.

**Fig. 5:**
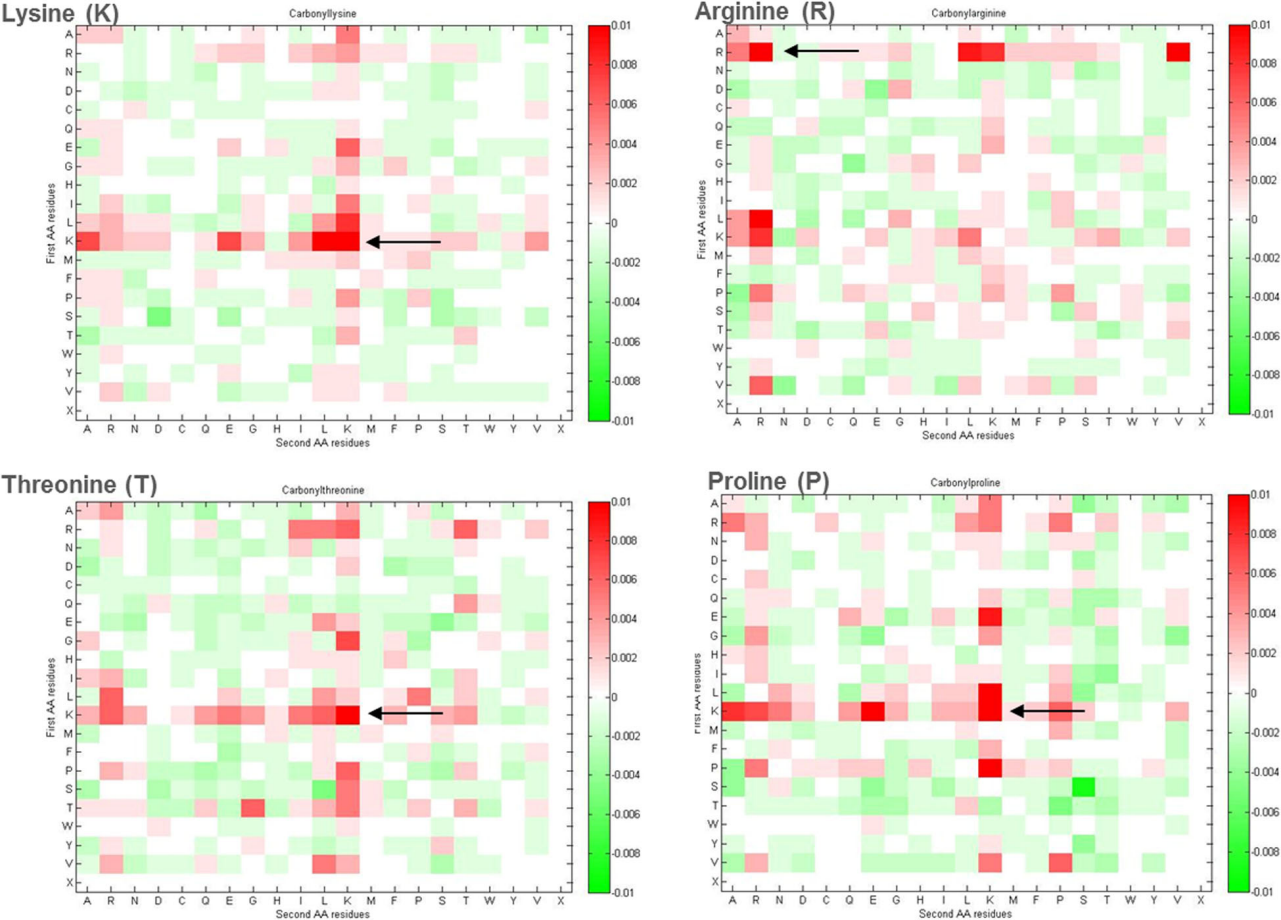
The frequency differences of 20 × 20 amino acid pairs between carbonylated sites and non-carbonylated sites of lysine, arginine, threonine and proline. The amino acid pair with red box indicates an over-representation in carbonylated sites (positive data) comparing to non-carbonylated sites (negative data); on the other hand, green box means an under-representation

In this study, the composition of amino acid pairs around carbonylation sites was further analyzed by means of selecting statistically significant dipeptides among the 400 amino acid pairs. The probability difference of 400 amino acid pairs between carbonylated and non-carbonylated sites were separately calculated on K, R, T and P residues. In the 20 × 20 matrix, amino acid pairs marked in red indicates an over-representation in carbonylation sites while amino acid pairs marked in green indicates an under-representation. As presented in Fig. 5, the dipeptides associated with K residue, such as KA, KE, KL, KK, EK and LK, are over-represented around carbonylated lysine residues. For the carbonylation sites on arginine, the dipeptides involved in R residue, including RR, RL, RK, RV, LR, and KR pairs, are observed to be over-represented around substrate sites. Additionally, it can also be observed that the K residues paired with other amino acids are over-represented around carbonylated sites on T and P residues. The *P*-value and the probability difference of each amino acid dipeptide is calculated as discussed previously. After ranking the dipeptides according to *P*-value, each amino acid pair having a *P*-value < 0.05 and a probability difference > 0.02 is considered as a statistically significant pair for the identification of protein carbonylation sites.

### Investigation of structural and physicochemical properties around carbonylation sites

**Fig. 6:**
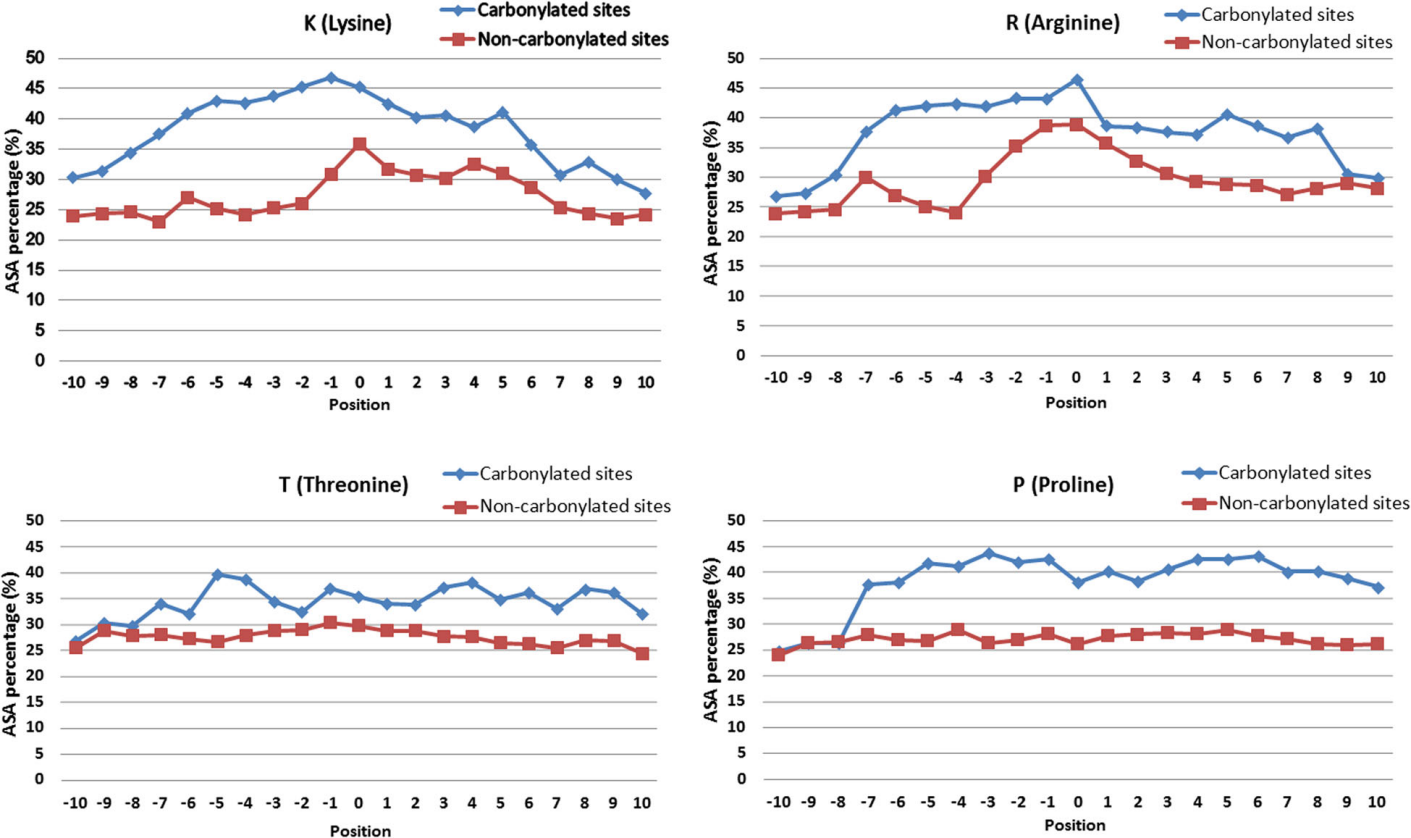
Comparison of the solvent-accessible surface area between carbonylated and non-carbonylated sites on K, R, T and P residues

It has been reported that a side-chain of amino acid that undergoes post-translational modification prefers to be accessible on the surface of a protein [66]. Although the tertiary structures of carbonylated proteins are limited, based on the prediction of ASA values by the RVP-Net tool, ASA was examined as an attribute for the identification of carbonylation sites. To explore how amino acids flanking the carbonylated and non-carbonylated sites might differ in their interaction with solvents, a comparison was performed using the average proportion of ASA based on the 21-mer window (-10 ~ +10). As shown in Fig. 6, amino acids surrounding the carbonylation sites exhibit higher ASA values compared to those around non-carbonylation sites. A strong evidence for hydrophilic preference at the carbonylated substrate sites was found because the average percentage of ASA values of the flanking residues was higher than non-carbonylated residues, especially for carbonylated K, R and P residues. Hence, hydrophilic amino acids flanking carbonylation sites might play functional roles for substrate sites specificity.

**Fig. 7:**
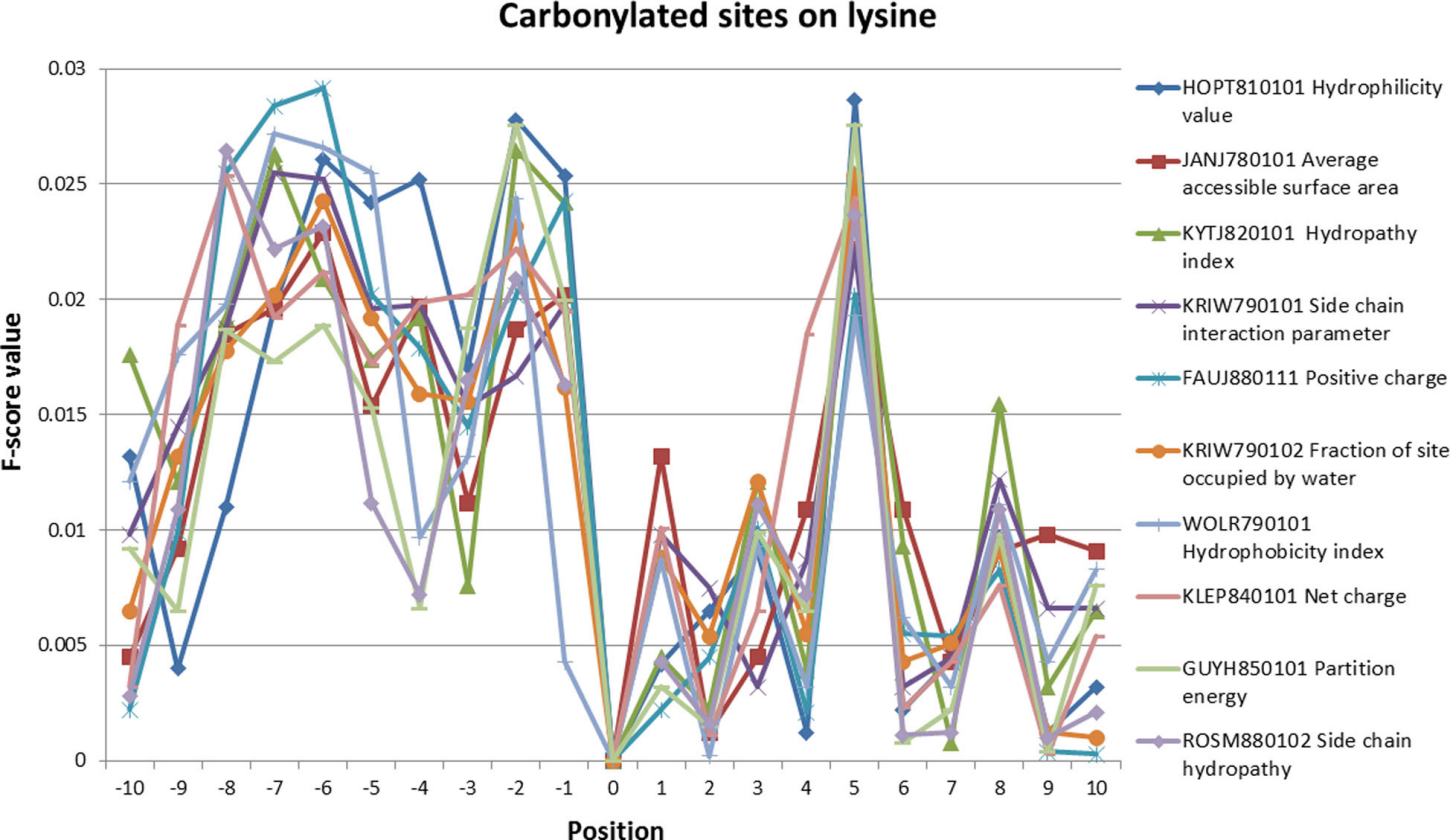
Top 10 physicochemical properties of carbonylated sites on lysine ranked by the average value of F-score measurement in 21-mer window

To further analyze the physicochemical property of carbonylation sites and adjacent amino acids, a total of 531 physicochemical properties were individually explored [67]. Figure 7 shows the top 10 physicochemical properties around the carbonylation sites on lysines, ranked by the average value of F-score measurement in 21-mer window (-10 ~ +10). The top ten physicochemical properties include Hydrophilicity value (HOPT810101), Average accessible surface area (JANJ780101), Hydropathy index (KYTJ820101), Side chain interaction parameter (KRIW790101), Positive charge (FAUJ880111), Fraction of site occupied by water (KRIW790102), Hydrophobicity index (WOLR790101), Net charge (KLEP840101), Partition energy (GUYH850101), and Side chain hydropathy (ROSM880102). This investigation reveals that the ten physicochemical properties contain higher F-score values at positions -8, -7, -6, -2 and +5, which have statistically significant difference between carbonylated and non-carbonylated K residues.

### Cross-validation performance of the models trained with various features

To examine what features can be adopted to construct the model that offers the best predictive performance (with balanced sensitivity and specificity) in the identification of carbonylated lysine, arginine, threonine and proline, three different classifiers, such as support vector machine (SVM), J48 decision tree, and random forest, were trained with various features and evaluated using five-fold cross-validation. In this study, totally seven kinds of training features, including amino acid sequence (AA), amino acid composition (AAC), amino acid pair composition (AAPC), positional weighted matrix (PWM), position-specific scoring matrix (PSSM), solvent-accessible surface area (ASA) and physicochemical properties (AAindex), were assessed based on a window size of 21-mer amino acids. In the prediction of carbonylated K residues, Table 2 shows that the SVM classifier could provide an overall better performance than DT and RF classifiers. Of the SVM models trained with individual features, that trained with PWM feature has highest sensitivity (0.748), accuracy (0.720) and MCC value (0.346), while that trained with physicochemical features (AAindex) gives a best specificity (0.720) in classifying between 206 carbonylated and 1166 non-carbonylated K residues. For carbonylated R residues, the SVM classifier also performs better than DT and RF classifiers. As presented in Table 3, the SVM model trained with PWM feature gives highest sensitivity (0.713) and MCC value (0.336), as well as the SVM model trained with AAindex feature provides the best specificity (0.726) and accuracy (0.721) in discriminating between 101 carbonylated and 504 non-carbonylated R residues. Additionally, Table 4 also shows that the SVM model trained with PWM feature yields best predictive performance in distinguishing 96 carbonylated and 488 non-carbonylated T residues, with the sensitivity of 0.688, the accuracy of 0.675 and MCC value of 0.274. However, the RF model trained with AAindex feature provides best specificity (0.676) in the prediction of carbonylated T residues. In the classification between 94 carbonylated and 412 non-carbonylated P residues, the SVM model trained with AAC feature could provide best sensitivity (0.745), while the SVM model trained with AAindex feature obtained highest specificity (0.752) and accuracy (0.743). Interestingly, the RF model trained with AAindex gives the best MCC value of 0.390, as shown in Table 5.

**Table 2:** Five-fold cross-validation results of the models trained with various features for classifying between 206 carbonylated and 1166 non-carbonylated lysine residues

Classifier	Training features	Sensitivity	Specificity	Accuracy	MCC
SVM	AA	0.680	0.643	0.649	0.235
	AAC	0.728	0.686	0.692	0.305
	AAPC	0.699	0.696	0.697	0.294
	PWM	*0.748*	0.715	*0.720*	*0.346*
	PSSM	0.704	0.686	0.689	0.288
	ASA	0.592	0.571	0.574	0.117
	AAindex	0.709	*0.720*	0.719	0.323
J48 DT	AA	0.534	0.557	0.554	0.066
	AAC	0.655	0.678	0.674	0.246
	AAPC	0.670	0.683	0.681	0.261
	PWM	0.689	0.674	0.676	0.267
	PSSM	0.621	0.660	0.655	0.207
	ASA	0.515	0.563	0.555	0.055
	AAindex	0.660	0.682	0.679	0.253
RF	AA	0.660	0.635	0.638	0.214
	AAC	0.704	0.686	0.689	0.288
	AAPC	0.709	0.703	0.704	0.307
	PWM	0.718	0.707	0.708	0.317
	PSSM	0.699	0.686	0.688	0.285
	ASA	0.583	0.583	0.583	0.119
	AAindex	0.709	0.717	0.716	0.319

The numbers marked with italicized font are the highest values in four measurements

**Table 3:** Five-fold cross-validation results of the models trained with various features for classifying between 101 carbonylated and 504 non-carbonylated arginine residues

Classifier	Training features	Sensitivity	Specificity	Accuracy	MCC
SVM	AA	0.614	0.603	0.605	0.163
	AAC	0.653	0.683	0.678	0.259
	AAPC	0.663	0.687	0.683	0.270
	PWM	*0.713*	0.718	0.717	*0.336*
	PSSM	0.624	0.685	0.674	0.239
	ASA	0.594	0.599	0.598	0.145
	AAindex	0.693	*0.726*	*0.721*	0.329
J48 DT	AA	0.554	0.603	0.595	0.119
	AAC	0.594	0.683	0.668	0.214
	AAPC	0.614	0.687	0.674	0.233
	PWM	0.614	0.675	0.664	0.222
	PSSM	0.554	0.665	0.646	0.169
	ASA	0.535	0.599	0.588	0.101
	AAindex	0.646	0.690	0.683	0.259
RF	AA	0.614	0.605	0.607	0.165
	AAC	0.634	0.683	0.674	0.244
	AAPC	0.653	0.683	0.678	0.259
	PWM	0.713	0.716	0.716	0.334
	PSSM	0.624	0.685	0.674	0.239
	ASA	0.594	0.599	0.598	0.145
	AAindex	0.693	0.724	0.719	0.327

The numbers marked with italicized font are the highest values in four measurements

**Table 4:** Five-fold cross-validation results of the models trained with various features for classifying between 96 carbonylated and 488 non-carbonylated threonine residues

Classifier	Training features	Sensitivity	Specificity	Accuracy	MCC
SVM	AA	0.625	0.615	0.616	0.180
	AAC	0.667	0.656	0.658	0.244
	AAPC	0.646	0.660	0.658	0.232
	PWM	*0.688*	0.672	*0.675*	*0.274*
	PSSM	0.656	0.656	0.656	0.236
	ASA	0.573	0.590	0.587	0.122
	AAindex	0.667	0.654	0.656	0.242
J48 DT	AA	0.604	0.594	0.596	0.148
	AAC	0.635	0.635	0.635	0.204
	AAPC	0.635	0.641	0.640	0.209
	PWM	0.625	0.637	0.635	0.198
	PSSM	0.604	0.598	0.599	0.151
	ASA	0.573	0.590	0.587	0.122
	AAindex	0.646	0.641	0.642	0.217
RF	AA	0.625	0.617	0.618	0.181
	AAC	0.656	0.652	0.652	0.233
	AAPC	0.646	0.652	0.651	0.225
	PWM	0.677	0.668	0.670	0.262
	PSSM	0.656	0.656	0.656	0.236
	ASA	0.583	0.594	0.592	0.133
	AAindex	0.656	*0.676*	0.673	0.254

The numbers makred with italicized font are the highest values in four measurements

**Table 5:** Five-fold cross-validation results of the models trained with various features for classifying between 94 carbonylated and 412 non-carbonylated proline residues

Classifier	Training features	Sensitivity	Specificity	Accuracy	MCC
SVM	AA	0.638	0.655	0.652	0.233
	AAC	0.713	0.716	0.715	0.347
	AAPC	0.646	0.728	0.713	0.309
	PWM	*0.745*	0.733	0.735	0.388
	PSSM	0.670	0.709	0.702	0.307
	ASA	0.585	0.607	0.603	0.151
	AAindex	0.702	*0.752*	*0.743*	0.375
J48 DT	AA	0.617	0.607	0.609	0.176
	AAC	0.638	0.631	0.632	0.212
	AAPC	0.638	0.636	0.636	0.216
	PWM	0.660	0.680	0.676	0.271
	PSSM	0.670	0.709	0.702	0.307
	ASA	0.574	0.583	0.581	0.123
	AAindex	0.649	0.709	0.698	0.290
RF	AA	0.628	0.660	0.654	0.229
	AAC	0.723	0.716	0.717	0.355
	AAPC	0.646	0.728	0.713	0.309
	PWM	0.734	0.733	0.733	0.380
	PSSM	0.660	0.704	0.696	0.294
	ASA	0.585	0.607	0.603	0.151
	AAindex	0.734	0.743	0.741	*0.390*

The numbers makred with italicized font are the highest values in four measurements

### Cross-validation performance of the models trained with hybrid features

In the investigation of predictive power of single features, the models trained with PWM usually provided better sensitivity than that trained with other features. On the other hand, the models trained with the selected physicochemical properties, top ten AAindices ranked by F-score measurement, could provide best specificity in discriminating carbonylation and non-carbonylation sites. In order to obtain better predictive power, moreover, the models trained with the combination of hybrid features were also evaluated by five-fold cross-validation. The combination of hybrid features was generated by combining two or more single features based on the mRMR-SFS feature-selection method, which incorporates the features sorted by mRMR scores. As presented in Additional file 5: Figure S3, a two-layered predictive model was generated from hybrid features based on mRMR-SFS feature selection. Using SVM as the classifier in Additional file 5: Figure S3), each selected feature was inputted to first-layered SVM for obtaining a feature-specific probability to form an input vector for generating second-layered SVM. In this investigation, the process of feature selection was terminated until predictive performance is not improved anymore. Finally, the models trained with the hybrid features and containing the best cross-validation performance were further evaluated using independent testing datasets.

For carbonylated K residues, Table 6 shows that the SVM model trained with the combination of PWM, AAC and AAindex features could improve the cross-validation performance with a sensitivity of 0.796, a specificity of 0.767, an accuracy of 0.711, and the MCC value of 0.432, when comparing to the SVM model trained with single PWM feature. In five-fold cross-validation of carbonylated R residues, the SVM model trained with the combination of PWM, AAindex and AAPC features provided the best MCC value (0.472), with the sensitivity of 0.782, the specificity of 0.798 and the accuracy of 0.795. In the prediction of carbonylation sites on T residues, the SVM model trained with the combination of PWM and AAindex features could reach the sensitivity of 0.750, the specificity of 0.795, the accuracy of 0.788 and the MCC value of 0.443. Additionally, the RF model trained with the combination of PWM, AAC and AAindex features could perform best in five-fold cross-validation of carbonylated P residues, which has the sensitivity of 0.787, the specificity of 0.777, the accuracy of 0.779 and the MCC value of 0.467.

**Table 6:** Five-fold cross-validation results of the models trained with the combination of hybrid features obtaining best predictive performance in training datasets

Residue	Classifier	Hybrid features	Sn	Sp	Acc	MCC
K	SVM	PWM + AAC + AAindex	0.796	0.767	0.771	0.432
R	SVM	PWM + AAindex + AAPC	0.782	0.798	0.795	0.472
T	SVM	PWM + AAindex	0.750	0.795	0.788	0.443
P	RF	PWM + AAC + AAindex	0.787	0.777	0.779	0.467

### Performance evaluation by independent testing datasets

In classifying between carbonylation and non-carbonylation sites, there is a possibility to overestimate the constructed model due to an overfitting of the training dataset. Thus to evaluate the real performance of the selected models with best cross-validation results, an independent testing dataset was manually extracted from seven research articles, which comprised experimentally verified carbonylation sites from multiple species. As given in Table 7, in classification between 78 carbonylated and 301 non-carbonylated K residues, the SVM model generated using the combination of PWM, AAC and AAindex features provides 0.641, 0.664, 0.659 and 0.252 for sensitivity, specificity, accuracy and MCC value, respectively. The SVM model trained with the hybrid features (PWM, AAindex and AAPC) could give a higher specificity (0.725) in discriminating between 67 carbonylated and 276 non-carbonylated R residues, with the sensitivity of 0.672, the accuracy of 0.714 and MCC value of 0.329. However, the SVM model trained using PWM and AAindex features provides a significantly higher sensitivity (0.755) in carbonylated T residues of the independent testing dataset, while the specificity is slightly low with the value of 0.605. The RF model trained with the the hybrid features (PWM, AAC and AAindex) also achieves a remarkably higher sensitivity (0.755) in carbonylated P residues of the independent testing dataset. In comparison with an existing prediction tool, the CarSPred could provide the best sensitivity (0.811) in carbonylated T residues of the independent testing dataset. Overall, our method performs better than CarSPred based on the independent testing performance.

**Table 7:** Comparison of independent testing results between our method and an available prediction tool (CarSPred)

Method	Residue	TP	FP	TN	FN	Sensitivity	Specificity	Accuracy	MCC
Our method	K	50	101	200	28	0.641	0.664	0.659	0.252
	R	45	75	201	22	0.672	0.725	0.714	0.329
	T	40	49	75	13	0.755	0.605	0.650	0.329
	P	62	105	199	20	0.756	0.658	0.679	0.342
CarSPred	K	44	112	189	34	0.564	0.631	0.617	0.161
	R	40	80	196	27	0.597	0.706	0.685	0.252
	T	43	74	50	10	0.811	0.403	0.525	0.208
	P	56	134	170	26	0.683	0.559	0.585	0.198

## Conclusion

Given the experimentally confirmed carbonylation sites, this study contributes a comprehensive characterization of substrate sites based on the composition of amino acids. The observation of position-specific amino acids composition indicated that the regions surrounding the carbonylation sites harbor a notable abundance of positively charged amino acids (K and R), especially for carbonylated K, R and P residues. This investigation suggested that the composition of amino acids may play a crucial role in discriminating between carbonylation and non-carbonylation sites. Additionally, the solvent accessibility and physicochemical properties were also examined in the characterization of carbonylated environment. A higher preference of solvent accessibility at the carbonylated residues was found because the average percentage of ASA values of the flanking residues was higher than non-carbonylated residues. Based on the F-score measurements on 531 physicochemical properties, top 10 AAindices were determined that have vital differences between carbonylation and non-carbonylation sites. According to the evaluation by five-fold cross-validation, among the predictive models trained from various features, the models trained with PWM feature had an overall higher sensitivity, while the models trained with AAindex feature achieved higher specificity. Furthermore, this investigation demonstrated that the model trained with hybrid features could provide better predictive performance than that trained with single feature. The independent testing results also revealed the effectiveness of the models trained with hybrid features in identifying protein carbonylation sites. In conclusion, this work not only characterized the substrate site preference, but also determined the best predictive model for the identification of carbonylation sites on K, R, T and P residues.

## Additional files

Additional file 1: Figure S1. Reaction process of protein carbonylation. (DOCX 623 kb)

Additional file 2: Table S1. Summary list of two previously published prediction tools of protein carbonylation sites. (DOCX 16 kb)

Additional file 3: Table S2. Data statistics of carbonylated sites obtained from literatures. (DOCX 18 kb)

Additional file 4: Figure S2. Flowchart of generating 400-dimensional PSSM vector by the PSSM profile. (DOCX 274 kb)

Additional file 5: Figure S3. Construction of two-layered predictive model using hybrid features based on mRMR-SFS feature selection. (DOCX 533 kb)
