# Supplementary figures and images for: Investigation and identification of protein carbonylation sites based on position-specific amino acid composition and physicochemical features
Source: BMC Bioinformatics. 2017 Mar 14;18(Suppl 3):66. doi: 10.1186/s12859-017-1472-8 (PMC5374553; doi:10.1186/s12859-017-1472-8)

**
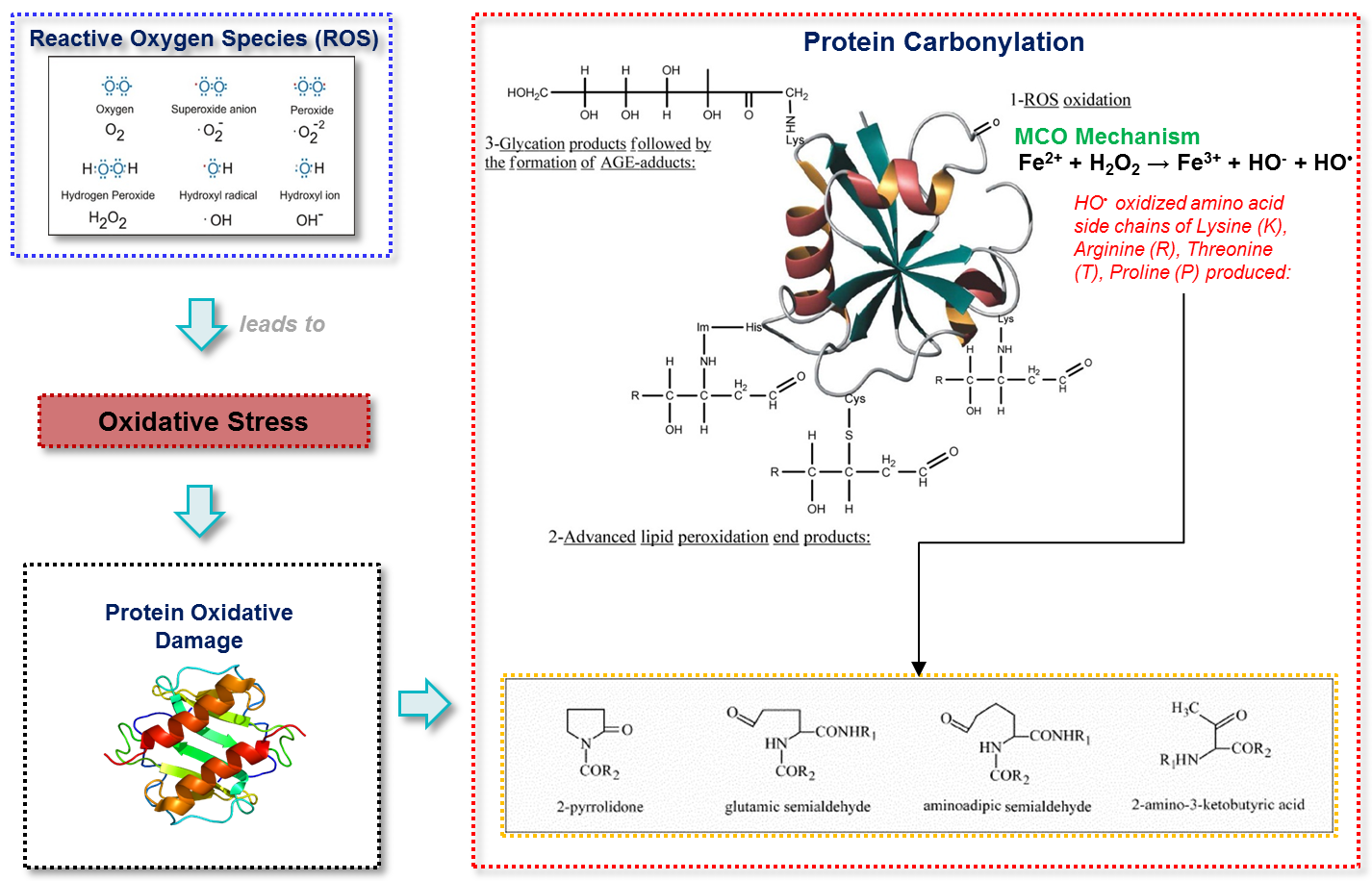
**

**Figure S1. Reaction process of protein carbonylation.**

Supplement: Supplementary file 1 — Reaction process of protein carbonylation. (DOCX 623 kb) [file 12859_2017_1472_MOESM1_ESM.docx]

**
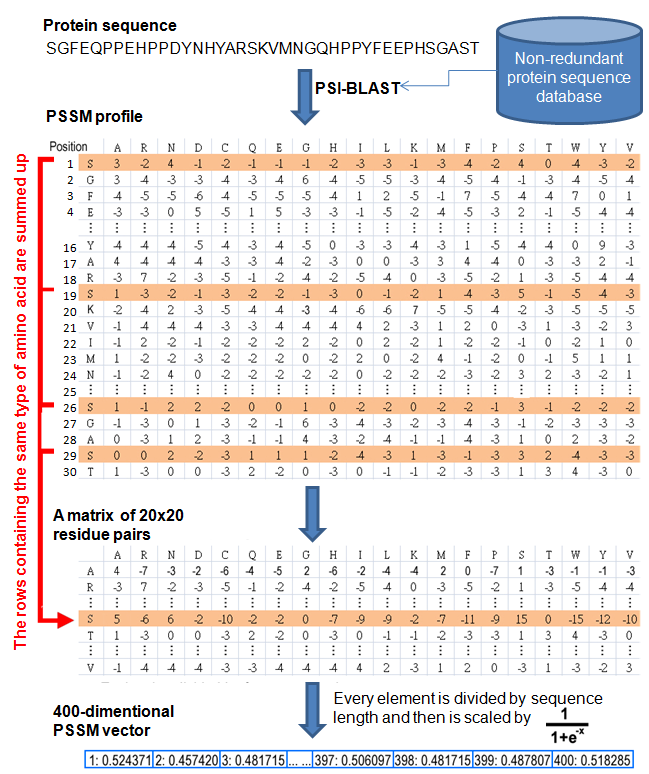
**

**Figure S2**. **Flowchart of generating 400-dimensional PSSM vector by the PSSM profile.**

Supplement: Supplementary file 4 — Flowchart of generating 400-dimensional PSSM vector by the PSSM profile. (DOCX 274 kb) [file 12859_2017_1472_MOESM4_ESM.docx]
