# Supplementary material for: Investigation and identification of protein carbonylation sites based on position-specific amino acid composition and physicochemical features
Source: BMC Bioinformatics. 2017 Mar 14;18(Suppl 3):66. doi: 10.1186/s12859-017-1472-8 (PMC5374553; doi:10.1186/s12859-017-1472-8)
Supplement: Supplementary file 2 — Summary list of two previously published prediction tools of protein carbonylation sites. (DOCX 16 kb) [file 12859_2017_1472_MOESM2_ESM.docx]

**Table S1. Summary list of two previously published prediction tools of protein carbonylation sites.**

| **Tool** | **Reference** | **Material** | **Method** | **K** | **R** | **T** | **P** | **K+R+T+P** | **Overall** |
| --- | --- | --- | --- | --- | --- | --- | --- | --- | --- |
| **CSPD** | Maisonneuve, et al. (2009) | A. thaliana, E. coli, B. subtilis, S. cerevisiae downloaded from NCBI ftp website (ftp://ftp.ncbi.nih.gov) | MALDI-TOF, LC nano-ESI MS/MS |  | **-** | **-** | **-** | **-** | Sn = 73%, Sp = 75%, Pre = 81% |
| **CarSPred** | Lv H, Han J, Liu J, Zheng J, Liu R, et al. (2014) | Experimental data in the 12 literatures | PSPAKSAAP, Increment of k-mer diversity, KNN scores, Physicochemical and biochemical properties | Acc =85.72% | Acc =85.95% | Acc =83.92% | Acc =85.72% | - | - |
