# Supplementary material for: Investigation and identification of protein carbonylation sites based on position-specific amino acid composition and physicochemical features
Source: BMC Bioinformatics. 2017 Mar 14;18(Suppl 3):66. doi: 10.1186/s12859-017-1472-8 (PMC5374553; doi:10.1186/s12859-017-1472-8)
Supplement: Supplementary file 3 — Data statistics of carbonylated sites obtained from literatures. (DOCX 18 kb) [file 12859_2017_1472_MOESM3_ESM.docx]

**Table S2. Data statistics of carbonylated sites obtained from literatures.**

| **Dataset** | **Resource** | **Organism** | **Number of carbonylated proteins** | **Number of carbonylated sites** | | | |
| --- | --- | --- | --- | --- | --- | --- | --- |
|  |  |  |  | K | R | T | P |
| **Training set** | Bollineni et al, 2014 [[21](#_ENREF_21)] | Human | 211 | 276 | 119 | 119 | 116 |
|  | Madian et al., 2011 [[19](#_ENREF_19)] | Human | 7 | 7 | 3 | 2 | 3 |
|  | Madian et al., 2010 [[20](#_ENREF_20)] | Human | 7 | 9 | 1 | 4 | 1 |
|  | Mirzaei & Regnier, 2006 [[18](#_ENREF_18)] | Human | 1 | 12 | 2 | 1 | 6 |
|  | Mirzaei & Regnier, 2006 [[17](#_ENREF_17)] | Human | 1 | 3 | 1 | 2 | 3 |
| **Independent testing set** | Kim et al., 2010 [[23](#_ENREF_23)] | Yeast | 6 | 6 | 3 | 2 | 2 |
|  | Maisonneuve et al., 2009 [[15](#_ENREF_15)] | *E. coli* | 23 | 7 | 12 | 21 | 38 |
|  | Mirzaei & Regnier, 2005 [[24](#_ENREF_24)] | Mouse/Rat | 5 | 1 | 4 | - | 1 |
|  | Mirzaei et al., 2008[[25](#_ENREF_25)] | Yeast | 12 | 10 | 4 | 6 | 5 |
|  | Mirzaei & Regnier, 2006a [[26](#_ENREF_26)] | Yeast | 54* | 35 | 21 | 12 | 20 |
|  | Mirzaei & Regnier, 2006b [[17](#_ENREF_17)] | Yeast | 26 | 12 | 4 | 10 | 8 |
|  | Mirzaei & Regnier, 2006c [[18](#_ENREF_18)] | Yeast | 92* | 46 | 42 | 11 | 30 |

**redundant proteins*
