# Supplementary material for: Investigation and identification of protein carbonylation sites based on position-specific amino acid composition and physicochemical features
Source: BMC Bioinformatics. 2017 Mar 14;18(Suppl 3):66. doi: 10.1186/s12859-017-1472-8 (PMC5374553; doi:10.1186/s12859-017-1472-8)
Supplement: Supplementary file 5 — Construction of two-layered predictive model using hybrid features based on mRMR-SFS feature selection. (DOCX 533 kb) [file 12859_2017_1472_MOESM5_ESM.docx]

**
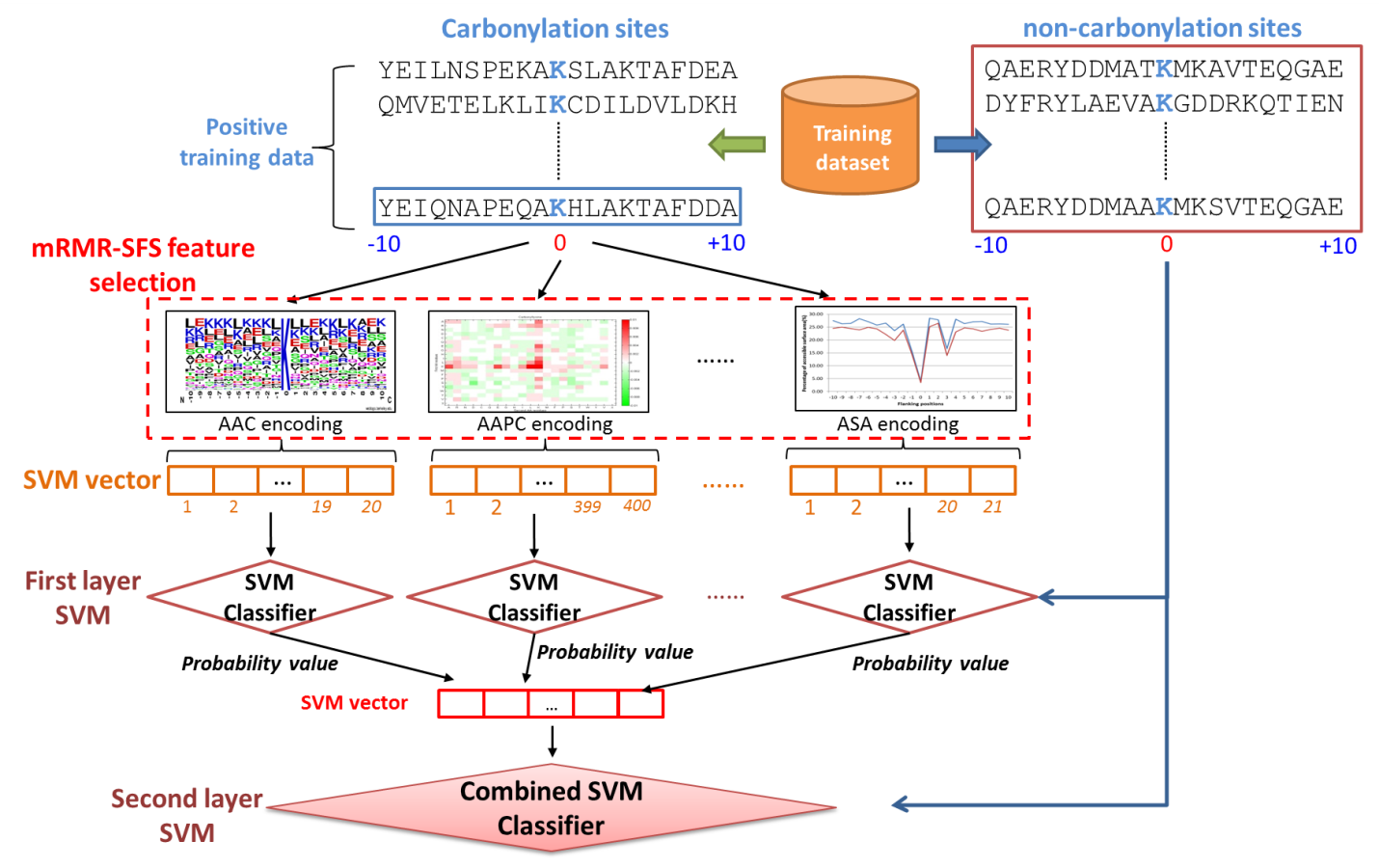
**

**Figure S3. Construction of two-layered predictive model using hybrid features based on mRMR-SFS feature selection.**
